# Salvage arthrodesis for failed total ankle arthroplasty: Clinical outcome and influence of method of fixation on union rate in 18 ankles followed for 3–12 years

**DOI:** 10.3109/17453671003628764

**Published:** 2010-03-31

**Authors:** H Cornelis Doets, Arthur W Zürcher

**Affiliations:** Department of Orthopedics, Slotervaartziekenhuis, Amsterdamthe Netherlands

## Abstract

**Background and purpose:**

Total ankle arthroplasty (TAA) has gained popularity in recent years. If it fails, however, salvage arthrodesis must be reliable as a rescue procedure. We therefore investigated the clinical, radiographic, and subjective outcome after salvage arthrodesis in a consecutive group of patients, and concentrated on the influence of the method of fixation on union rate and on salvage in inflammatory joint disease.

**Patients and methods:**

Between 1994 and 2005, salvage arthrodesis was performed on 18 ankles (18 patients). Diagnosis was inflammatory joint disease (IJD) in 15 cases and osteoarthritis (OA) in 3. Tibio-talar fusion was performed in 7 ankles, and tibio-talocalcaneal fusion in 11. Serial radiographs were studied for time to union. Clinical outcome at latest follow-up was measured by the AOFAS score, the foot function index (FFI) and by VAS scores for pain, function, and satisfaction.

**Results:**

Blade plates were used in 7 ankles (4 IJD, 3 OA); all united. Nonunion developed in 7 of the 11 rheumatic ankles stabilized by other methods. 11 patients (8 fused ankles, 3 nonunions) were available for clinical evaluation. Their mean AOFAS score was 62 and mean overall FFI was 70. VAS score for pain was 20, for function 64, and for satisfaction 74. The scores were similar in united and non-united ankles.

**Interpretation:**

Blade plate fixation is successful in salvage arthrodesis for failed TAA. A high nonunion rate was found after salvage ankle arthrodesis in IJD with other methods of fixation. Clinical results were fair to good.

## Introduction

Total ankle arthroplasty (TAA) with use of a mobile-bearing design is currently seen as an alternative for arthrodesis in the treatment of the painful arthritic ankle. Several reports exist on the medium-term to long-term results with use of such third-generation designs, showing a satisfactory survival at 8 years of between 84% and 94% (Buechel et al. 2003, Doets et al. 2006, Wood et al. 2008). If TAA fails, however, salvage ankle arthrodesis has to be a reliable rescue procedure if implant exchange is not feasible.

Some early studies have addressed the results of salvage arthrodesis of failed first-generation constrained two-component designs. The number of ankles treated for inflammatory joint disease (IJD, mostly rheumatoid arthritis) in these studies varied. Stauffer (1982) was the first to report on the results of salvage arthrodesis after failed TAA. He found a solid fusion in all 17 ankles (underlying diagnosis not given). Groth and Fitch (1987) reported successful salvage arthrodesis in 11 osteoarthritis (OA) cases. Kitaoka and Romness (1992) included 10 cases of rheumatoid arthritis (RA) in their series of 37 ankles. Union was achieved in 33 ankles. Only the series reported by Carlsson et al. (1998) included a relatively large number of patients with RA: 16 of 21 ankles. In their study, 8 patients had no fusion at the first attempt, 7 of them having RA.

Recently, some studies have been published on the conversion to arthrodesis for failed mobile-bearing TAA. Hopgood et al. (2006) found good results in OA ankles, and in RA ankles treated with a retrograde nail. However, RA ankles stabilized by screw fixation failed to heal. Culpan et al. (2007) published successful results of fusion for failed TAA in 16 ankles (mostly OA). Anderson et al. (2005c) reported on 16 salvage fusions in RA ankles with use of a retrograde nail and either allograft or autologous bone. 11 healed at the first attempt, and 2 others healed after repeat arthrodesis.

The aim of this study was to determine whether salvage arthrodesis could be an adequate rescue procedure after failed mobile-bearing TAA, with a special focus on rheumatic ankles. Secondary questions were: which salvage arthrodesis techniques were successful, and what was the subjective outcome.

## Patients and methods

This study protocol was approved by the local ethics committee on December 5, 2005 (registration number U/228/0518), and all patients seen at follow-up gave their informed consent. Total ankle arthroplasty with use of a mobile-bearing design was first used at our institution in 1988, and until 2000 it was mainly carried out in patients suffering from IJD (Doets et al. 2006). All TAA patients at our institution enter a prospective study protocol. During the study period, conversion to a tibio-talar or tibio-talocalcaneal arthrodesis was the standard surgical treatment for failed TAA.

Between 1994 and 2005, 18 patients (18 ankles) underwent a salvage arthrodesis for failed TAA (Table). Mean age at the time of TAA was 55 (27–76) years and mean interval between TAA and the salvage arthrodesis was 4 (0.2–13) years. There were 15 patients with IJD (mostly RA) and 3 patients with OA. At the time of the salvage procedure, 9 hindfeet in the rheumatic population were ankylosed, either by a formal surgical fusion or by having occurred spontaneously.

**Table:** Demographic, perioperative, and outcome data on all salvage procedures

A	B	C	D	E	F	G	H	I	J	K	L	M
1	F	27	JIA	LCS	Loosening	161	Ankylosis	TTC	Screws	Allo	Nonunion	–
2 **^a^**	F	49	RA	LCS	Instability	83	Arthritis	TT	Blade plate	AuP	3	–
3 **^a^**	M	63	RA	LCS	Deformity	103	Normal	TT	Blade plate	AuL	4	–
4 **^a^**	F	58	RA	LCS	Loosening	115	Fusion	TTC	K-wires	Allo	Nonunion	–
5 **^a^**	F	66	RA	LCS	Deformity	25	Ankylosis	TTC	Screws	AuP	10	–
6 **^a^**	M	51	RA	LCS	Deformity	51	Fusion	TTC	Blade plate	AuL	2	–
7	F	60	RA	BP	Deformity	44	Fusion	TTC	Nail	AuL	16	Nail dynamization
8	F	43	RA	BP	Postop. malleolar fracture	42	Fusion	TTC	Nail	AuFH (deep-frozen)	Nonunion	–
9 **^a^**	F	71	RA	BP	Instability	20	Fusion	TTC	Screws	AuP	Nonunion	Repeat arthrodesis
10	F	41	RA	BP	Infection	4	Arthritis	TT	Screws	AuL	3	–
11	M	51	RA	BP	Loosening	40	Fusion	TTC	Nail	AuP	Nonunion	2 Repeat arthrodeses
12	F	73	RA	BP	Loosening	50	Arthritis	TTC	Nail	AuP	Nonunion	Repeat arthrodesis
13	F	45	RA	BP	Loosening	31	Ankylosis	TTC	Nail	AuL	9	Nail removal
14	M	55	OA	BP	Pain	61	Normal	TT	Blade plate	AuL	2	–
15 **^b^**	F	76	RA	BP	Wound dehiscence	2	Arthritis	TTC	Nail	None	Nonunion	Repeat arthrodesis
16	F	47	OA	BP	Pain	39	Normal	TT	Blade plate	AuL	4	–
17	F	46	NsO	BP	Wound dehiscence	6	Normal	TT	Blade plate	AuP	4	Plate removal
18	F	61	OA	CCI	Intraop. malleolar fracture	3	Normal	TT	Blade plate	Allo + AuL 10	–	
A Case no.												
**^a^** Patients who died before 2008 with no clinical scores at follow-up.												
**^b^** Patient wheelchair-bound; no clinical scores at follow-up.												
B Sex												
C Age at TAA												
D Diagnosis												
JIA: juvenile idiopathic arthritis;												
RA: rheumatoid arthritis;												
NsO: non-specific oligoarthritis;												
OA: osteoarthritis.												
E Prosthesis												
LCS = New Jersey low contact stress;												
BP: Buechel-Pappas;												
CCI: ceramic-coated implant.												
F Failure scenario												
G Interval since TAA (months)												
H Subtalar joint												
I Type of fusion												
TT: Tibio-talar;												
TTC: tibio-talocalcaneal.												
J Fixation												
K Type of bone graft												
Allo: allograft;												
AuL: autologous bone graft harvested locally;												
AuP: autologous bone graft from pelvis;												
AuFH: autologous bone graft from deepfrozen femoral head.												
L Time to fusion (months)												
M Secondary surgery												

### Surgical technique

16 of the 18 salvage procedures were done by 2 experienced foot and ankle surgeons, and 2 other surgeons who were experienced in the field of rheumatoid arthritis surgery each performed 1 procedure.

The fusion technique applied depended on the following factors: condition of the subtalar joint at the time of surgery, quality of the local bone, and the extent of local bone loss. Blade plates (either an AO humeral plate or an AO child hip plate; Synthes GmbH, Solothurn, Switzerland) were used in 6 tibio-talar arthrodeses and 1 tibio-talocalcaneal arthrodesis. The plates were implanted either at the anterior or the lateral aspect of the ankle. Compression at the arthrodesis site was applied with an AO compression device. Blade plate fixation was our preferred technique in the presence of a normal subtalar joint, as rigid fixation could be obtained without interference with the subtalar joint in such cases. In 6 ankles, an intramedullary locking nail was used to stabilize the ankle, implanted in a retrograde fashion. In 4 ankles, 2–3 compression screws were used, and 1 ankle in an elderly RA patient with severe osteopenia was stabilized by multiple K-wires.

In 14 ankles, a cancellous autologous bone graft was used to fill osseous defects, mostly harvested locally. In 3 ankles, morselized allograft bone was used; in 1 of these, it was combined with autologous bone.

### Clinical evaluation

Medical charts of the failed ankle arthroplasties were reviewed in detail for patient characteristics, reason for failure of the prosthesis, fusion technique, and for any postoperative complication or reoperation. At the time of the final evaluation in 2008, the following 3 clinical instruments were used to assess the clinical result of all ankles in follow-up:

The ankle-hindfoot score as developed by the American Orthopaedic Foot and Ankle Society (AOFAS). It is a 100-point score, consisting of both subjective and objective clinical parameters (Kitaoka et al. 1994). The maximum attainable AOFAS score is 89 points after a tibio-talar arthrodesis, and after a tibio-talocalcaneal arthrodesis it is 86 points.

The Foot Function Index with verbal rating scales (FFI-5pt). This is a self-administered questionnaire that pertains to complaints in the foot and ankle during daily life. The scale consists of 3 subscales: limitation (5 items), pain (9 items), and disability (9 items). The items of the FFI-5pt are rated on a 5-point scale. To calculate the subscale scores, the item scores are summed up, divided by the maximum possible sum of the item scores, and then multiplied by 100 in order to calculate the definitive subscale scores. The total score is the mean of the subscale scores and ranges from 0 to 100. Contrary to other systems, higher scores indicate more limitation, pain, and disability. A Dutch version of the FFI-5pt has been validated (Kuyvenhoven et al. 2002).

Visual analog scales (VAS) with a scale from 0 to 100, to score pain (where 0 means no pain), limitation of function (where 0 represents complete limitation), and satisfaction of the treatment result (where 0 means very dissatisfied).

### Radiographic evaluation

For the radiographic evaluation, the serial radiographs were evaluated for the time to fusion (at the first or at the second or third attempt) and for the alignment of the fused ankle in the coronal and the sagittal plane. Osseous union was defined as the formation of trabeculae across the line of arthrodesis. This radiographic evaluation was done by a radiologist (JPK) who was not involved in the care of these patients.

### Statistics

Two-sided Fisher's exact test was used to determine the influence of fixation method (blade plate vs. nail or screws) in the IJD population. 95% confidence intervals (CIs) were calculated and Fisher's exact test was done using SPSS software version 14.

## Results

In 2008, at the final follow-up, the mean follow-up time of all 18 salvaged ankles was 7.3 (3–12) years.

### Union rate and method of fixation (Table)

11 of the 18 ankles healed after a first attempt. Mean time to solid fusion in this group was 6.3 (2–16) months (CI: 3.5–9.1). All 7 nonunions occurred in the group of 15 patients with IJD. 4 nonunions underwent a second-attempt salvage arthrodesis, resulting in union in 2. 1 ankle failed to unite after a third attempt. The reoperations are described below in detail.

**Figure 1.**
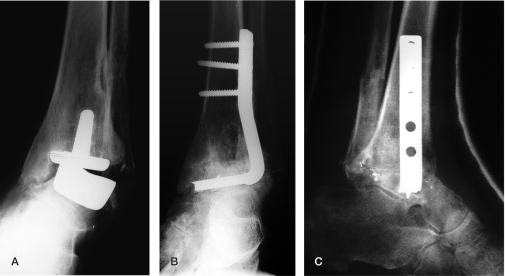
A 63-year-old man (case 3) with long-standing RA and a preoperative varus deformity of the ankle of 20 degrees. A. After implantation of an LCS prosthesis. There is a persistent varus deformity and edge-loading of the prosthesis. B and C. After conversion to tibiotalar arthrodesis. The arthrodesis was stabilized by a humeral blade plate, implanted at the lateral side. Debris originating from the edge-loading of the metallic components is visible at the arthrodesis site.

**Figure 2.**
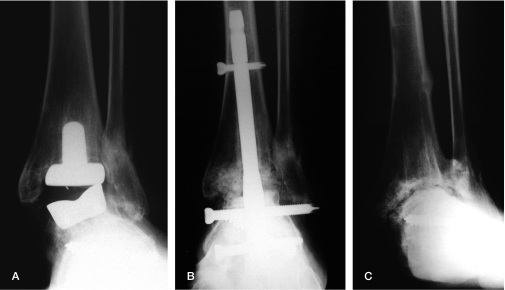
A 43-year-old woman (case 8) with RA had a Buechel-Pappas prosthesis implanted. She had a preoperative valgus deformity of 10 degrees. A. 3 years postoperatively, when, due to a malleolar insufficiency fracture, a recurrent valgus deformity with edge-loading of the prosthesis had developed. B. A tibio-talocalcaneal arthrodesis was performed, stabilized by a retrograde intramedullary locking nail. C. 2 years after the salvage procedure, the nail had been removed. 6 years later, there was a fibrous nonunion. The ankle was fairly asymptomatic and did not require a second-attempt procedure.

All 7 ankles (4 IJD and 3 OA) in which a blade plate was used united at the first attempt (Figure 1). In contrast, 4 out of 6 first-attempt procedures stabilized with a retrograde nail (Figure 2), 2 out of 4 ankles stabilized with screws, and the ankle stabilized by K-wires developed a nonunion. The difference in union rate between the rheumatoid ankles stabilized by either a retrograde nail or screws and by a blade plate was not statistically significant (p = 0.08).

7 of 8 rheumatoid ankles in which a locally harvested bone graft was used healed after a first-attempt salvage. 3 of 5 rheumatoid ankles in which an autologous bone graft from the iliac crest was used healed after first-attempt salvage arthrodesis. In 2 rheumatoid ankles, only allograft was used; both ended as a nonunion.

In the 3 osteoarthritic ankles, all treated by blade plate fixation and additional bone graft, osseous union was seen at 2, 4, and 10 months (see Table).

### Complications and reoperations

1 patient (no. 13) required nail extraction at 4 months because of a low-grade infection (due to secondary perforation of the skin by the tibial locking screw). As the ankle was clinically stable, it was immobilized in a brace for 2 months and no further surgical treatment has been necessary. Another patient (no. 17), stabilized by a blade plate, also required hardware extraction at 4 months because of a low-grade infection. Although this ankle was solidly fused, further debridement and soft-tissue procedures became necessary at follow-up because of a small persistent fistula. A delayed union developed in 1 patient (no. 7) stabilized with an intramedullary locking nail. It eventually united 16 months postoperatively, 6 months after dynamization of the nail. Seven patients with a successful first-attempt salvage arthrodesis required hardware removal because of symptoms from the material.

In 7 patients, all suffering from IJD, a nonunion developed after the first attempt to fuse the ankle. 4 of these patients underwent repeat arthrodesis, whereas 3 refused further surgery. 2 of the latter patients had a stiff and painless fibrous nonunion. Details of the 4 repeat arthrodeses are as follows.

Patient 9 underwent a salvage arthrodesis stabilized by screw fixation. Her hindfoot had already been fused prior to the TAA. 4 months after the initial salvage, a re-arthrodesis with use of a blade plate had to be done for instability, resulting in a solid fusion. However, some months after implant removal a spontaneous talar neck fracture developed, for which re-osteosynthesis with use of a retrograde nail had to be carried out.

Patient 11 developed mechanical loosening 3 years after a 2-stage revision TAA for a deep infection. The arthrodesis, stabilized by a retrograde nail, ended in a fibrous nonunion. A second attempt with a blade plate was made 6 years after the first salvage procedure. It was complicated by a wound dehiscence and an early deep infection, for which multiple debridements had to be carried out. Eventually, a third-attempt salvage with a compression intramedullary locking nail was performed. However, this ankle again failed to unite.

Patient 12 developed mechanical loosening 3 years after her TAA. Her ipsilateral hip had become ankylosed long before the ankle replacement in a position of slight flexion and significant external rotation. An arthrodesis with use of a retrograde nail was done. After material extraction and debridement for an infected nonunion, a re-arthrodesis with use of an external fixator and autologous bone graft was done. Despite all efforts, a nonunion remained the end result. The deformed hip probably contributed to both the early mechanical loosening of the TAA and to the nonunion of the rescue procedures. Total hip arthroplasty was offered, but the patient refused.

Patient 15 was a failed primary TAA due to a severe wound dehiscence with open joint. A 1-stage salvage arthrodesis, stabilized by an intramedullary locking nail, was done 2 months after the index surgery. This resulted in a low-grade infected nonunion, for which a 2-stage re-arthrodesis with use of an external fixator was carried out. The ankle united, but was complicated by a septic arthritis of the talonavicular joint, requiring subsequent surgery.

### Clinical outcome and radiographic alignment

The clinical outcome at the latest follow-up could be assessed in 11 patients (6 patients had died: cases 2, 3, 4, 5, 6 and 9; and 1 patient, case 15, was wheelchair-bound due to generalized arthritic disease and judged herself unable to give a reliable subjective outcome). Mean interval since the first-attempt salvage arthrodesis in this group was 7.8 (3.1–12) years. The mean AOFAS score was 62 (38–89) (CI: 54–71), and the mean overall FFI score was 70 (62–78). The mean VAS for pain was 20 (CI: 7.2–33), the mean VAS for function was 64 (CI: 45–84), and the mean VAS for satisfaction was 74 (CI: 61–87). The 4 ankles in follow-up with a persistent nonunion had subjective results similar to those for the fused ankles.

The radiographic mean sagittal angle of the 11 ankles that healed after first-attempt salvage was 6 degrees of equinus (CI: 0.6–11). 8 ankles had a neutral alignment in the coronal plane (0–5 degrees of valgus), 2 ankles had healed in slight varus, and 1 ankle had healed in 15 degrees of valgus.

## Discussion

Salvage arthrodesis should be a reliable treatment option if TAA fails and revision by implant exchange is impossible due to bone loss, deformity, or infection. Several reports have shown that salvage arthrodesis for failed ankle replacement has a mean fusion rate of 74–100% (Stauffer 1982, Groth and Fitch 1987, Kitaoka and Romness 1992, Carlsson et al. 1998, Anderson et al. 2005c, Hopgood et al. 2006, Kotnis et al. 2006, Culpan et al. 2007). These results are similar to the success rate of arthrodesis for end-stage ankle arthritis (primary ankle arthrodesis). In a meta-analysis, Haddad et al. (2007) described a 90% union rate after primary ankle arthrodesis. In general, the success rate of primary ankle arthrodesis in IJD is somewhat inferior. Dereymaker et al. (1998) had 5 nonunions in a series of 14 ankles. Anderson et al. (2005b) had 9 nonunions in 35 ankles stabilized by screw fixation. Better results were published from the same institution when a retrograde nail had been used: 1 nonunion out of 26 tibiotalocalcaneal fusions (Anderson et al. 2005a). The largest series of ankle fusions in IJD, performed through a transfibular approach, was published by Mäenpää et al. (2001) from the Rheumatism Foundation Hospital in Finland. In their series of 130 ankles, 90% united. They concluded that ankle arthrodesis in IJD is a demanding procedure, that the operation should be performed by an experienced surgeon, and that correction of malalignment and the use of bone grafts are of crucial importance for fusion.

In our series, nonunion only occurred in the rheumatoid ankles. This emphasizes the fact that it seems to be more difficult to obtain solid union of both primary and salvage ankle arthrodesis in IJD patients. 2 of 4 second-attempt procedures were successful, and 2 of the 3 primary nonunions had developed a stable fibrous nonunion. Clinical outcome of salvage arthrodesis was relatively good, with fair to good FFI and AOFAS scores, and mostly good VAS pain and satisfaction scores. In view of the fact that the clinical scores of the united and nonunited ankles were similar, a fibrous nonunion does not appear to be a disastrous event. It should be realized, however, that clinical scores were obtained from only 11 cases.

In our hands, in salvage ankle arthrodesis, blade plate fixation was the most successful technique. With the small numbers available, no statistically significant differences could be found in the rheumatic subgroup in comparison with more commonly used methods of fixation. The advantage of blade plate fixation is that a stable fixation can be obtained and that no hardware is present inside the arthrodesis site. Good results with blade plate fixation for tibio-calcaneal and tibio-talocalcaneal arthrodeses have been published (Myerson et al. 2000, Hanson and Cracchiolo 2002). The good stability of blade plate fixation was also shown in the biomechanical study by Chiodo et al. (2003). They found greater stability of tibio-talocalcaneal arthrodeses stabilized by a blade-plate-and-screw construct than with a retrograde intramedullary locking nail. As far as we know, no results have been published on blade plate fixation for tibio-talar arthrodesis.

In conclusion, in osteoarthritis the union rate of salvage ankle arthrodesis is good, and comparable to the outcome of primary ankle arthrodesis. In rheumatoid ankles, both primary arthrodesis and salvage arthrodesis are demanding procedures, and they should probably best be done by experienced surgeons in specialized centers. Stabilization by a blade plate seems to be a promising technique for salvage ankle arthrodesis.
